# Antimicrobial‐associated encephalopathy due to ampicillin

**DOI:** 10.1002/ccr3.8665

**Published:** 2024-04-02

**Authors:** Naoya Mizutani, Tsuneaki Kenzaka

**Affiliations:** ^1^ Department of Internal Medicine Hyogo Prefectural Tamba Medical Center Tamba Hyogo Japan; ^2^ Division of Community Medicine and Career Development Kobe University Graduate School of Medicine Kobe Hyogo Japan

**Keywords:** ampicillin, antimicrobial‐associated encephalopathy, beta‐lactams, penicillin, seizures

## Abstract

Because the β‐lactam ring has a molecular structure similar to that of gamma‐aminobutyric acid (GABA) neurotransmitters, it binds to GABA A receptors and inhibits GABAergic transmission, causing AAE. The possibility of antimicrobial‐associated encephalopathy should be considered in cases of neurological or psychiatric symptoms after initiating an antimicrobial regimen.

An 84‐year‐old man with a history of mitral and aortic valve replacement surgery for infective endocarditis presented with a complaint of fever that had persisted for 2 weeks. *Enterococcus faecalis* was detected in blood culture. The source of infection was unknown, and ampicillin was initiated after identifying the causative organism. Myoclonus of the right forearm appeared 4 days after treatment (Video S1). Head MRI (Figure 1) and EEG (Figure 2) scans revealed no abnormalities. Ampicillin was changed to daptomycin, and the myoclonus improved the day after. Based on the course of the disease, a diagnosis of antimicrobial‐associated encephalopathy caused by ampicillin was made.

**FIGURE 1:**
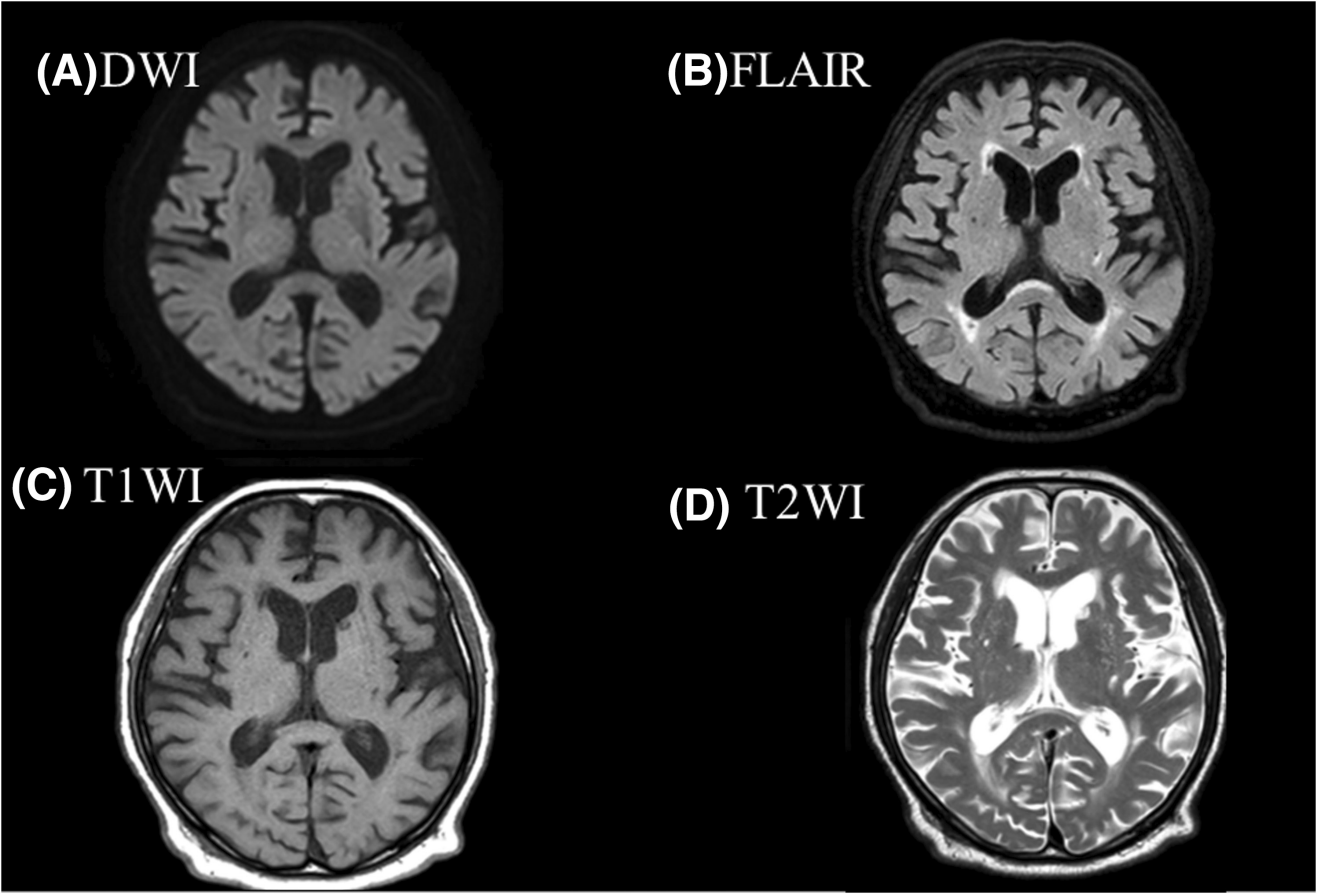
Head magnetic resonance imaging. (a) DWI, diffusion‐weighted imaging; (b) FLAIR, fluid‐attenuated inversion recovery; (c) T1WI, T1‐weighted image; (d) T2WI, T2‐weighted image. There is no image of a stroke.

**FIGURE 2:**
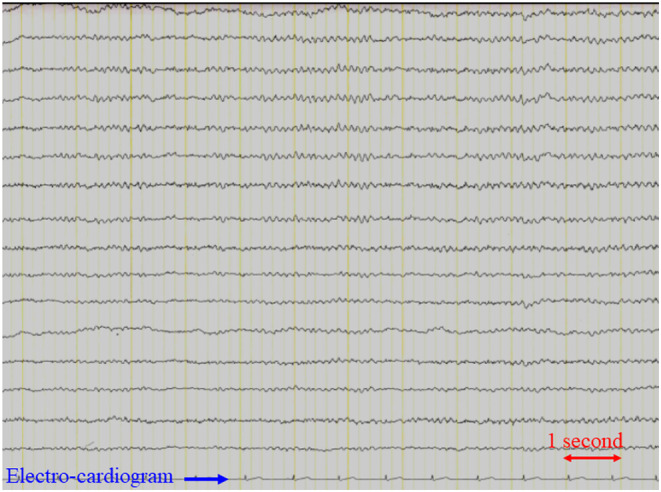
Electroencephalograph. Electroencephalograph was recorded at rest, awake, and with eyes closed. It shows a slow wave of approximately 7–8 Hz. No abnormal brain waves that would suggest epilepsy were detected.

## AUTHOR CONTRIBUTIONS

**Naoya Mizutani:** Conceptualization; investigation; methodology; visualization; writing – original draft. **Tsuneaki Kenzaka:** Conceptualization; investigation; methodology; supervision; validation; writing – original draft; writing – review and editing.

## FUNDING INFORMATION

None of the authors have any financial interests to disclose.

## CONFLICT OF INTEREST STATEMENT

None of the authors have any have any conflicts of interest to declare.

## CONSENT

Written informed consent was obtained from the patient to publish this report in accordance with the journal's patient consent policy.

## Supporting information


Video S1.

## Data Availability

Data available on request from the authors.
